# Evidence Lacking for Endemic Chagas Disease in the United States (Response)

**DOI:** 10.3201/eid3205.260617

**Published:** 2026-05

**Authors:** Norman L. Beatty, Gabriel L. Hamer, Bernardo Moreno-Peniche, Bonny Mayes, Sarah A. Hamer

**Affiliations:** University of Florida College of Medicine, Gainesville, Florida, USA (N.L. Beatty); Emerging Pathogens Institute, University of Florida, Gainesville (N.L. Beatty); Texas A&M University, College Station, Texas, USA (G.L. Hamer); University of California, Berkeley, California, USA (B. Moreno-Peniche); Texas Department of State Health Services, Austin, Texas, USA (B. Mayes); Texas A&M University College of Veterinary Medicine and Biomedical Sciences, College Station (S.A. Hamer)

**Keywords:** Chagas disease, *Trypanosoma cruzi*, triatomine, parasites, vector-borne infections

**In Response:** We thank Cantey et al. (*1*) for their work and acknowledge our shared perspective that awareness for Chagas disease in the United States must be raised.

## References

[R1] Cantey PT, Hast M, Chancey RJ, Mongomery SP. Evidence Lacking for Endemic Chagas Disease in the United States. Emerg Infect Dis. 2026;32:830–831.10.3201/eid3205.25184042116553

